# Rab22a-NeoF1: a promising target for osteosarcoma patients with lung metastasis

**DOI:** 10.1038/s41392-020-00273-w

**Published:** 2020-08-24

**Authors:** Kai Xie, Xinyi Zhang, Yongguang Tao

**Affiliations:** 1 Key Laboratory of Carcinogenesis and Cancer Invasion, Ministry of Education, Department of Pathology, Xiangya Hospital, Central South University, Changsha, 410078 Hunan China; 2 Hunan Key Laboratory of Early Diagnosis and Precision Therapy, Department of Thoracic Surgery, The Second Xiangya Hospital, Central South University, Changsha, 410011 Hunan China; 3 Department of Neurosurgery, Xiangya Hospital, Central South University, Changsha, 410008 Hunan China; 4 Department of Cardiovascular Medicine, Xiangya Hospital, Central South University, Changsha, 410008 Hunan China; 5 Department of Cardiovascular Medicine, Third Xiangya Hospital, Central South University, Changsha, 410013 Hunan China; 6 NHC Key Laboratory of Carcinogenesis (Central South University), Cancer Research Institute and School of Basic Medicine, Hunan Key Laboratory of Oncotarget Gene, Central South University, Changsha, 410078 Hunan China

**Keywords:** Metastasis, Bone cancer

**Fig. 1:**
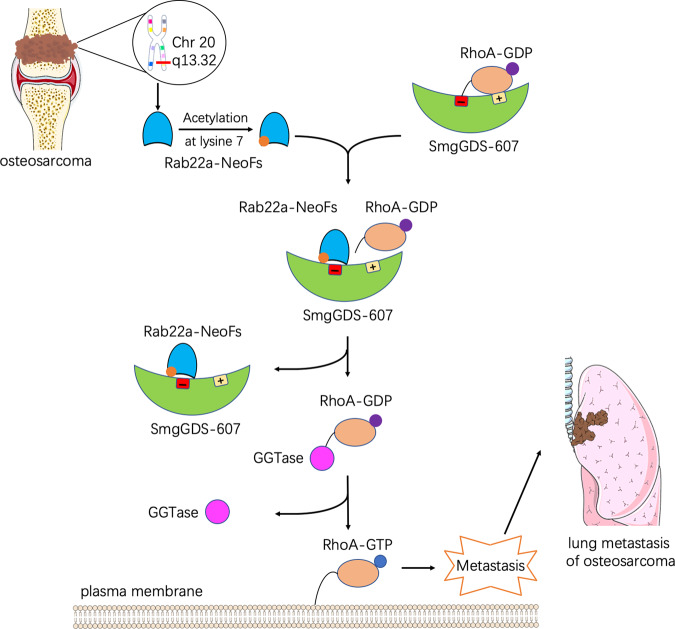
Fusion proteins, designated as Rab22a-NeoFs (Rab22a-NeoF1–6), are encoded by exon–intron fusion genes located at chromosome 20 from osteosarcoma patients with lung metastasis. Acetylation of Rab22a-NeoF1 at lysine 7 is implicated in the association between Rab22a-NeoF1 and SmgGDS-607. Rab22a-NeoF1 constitutively binds to the negatively charged region of SmgGDS-607 to diminish the interaction between SmgGDS-607 and RhoA. Then, GDP–GTP exchange on RhoA is accelerated and more RhoA–GTP is transported to the plasma membrane to promote lung metastasis of osteosarcoma

Osteosarcoma is the most common primary malignant bone tumor among adolescents and children, with high level of lung metastasis and poor prognosis. The treatment of metastatic cases remains a challenge in the clinic, leading to the dramatically decreased survival rate. However, the molecular mechanisms are still unclear. A recent article by Kang et al. demonstrated how chromosomal translocations in osteosarcoma facilitated lung metastasis, and shed new light on its therapeutic strategies^1^ (Fig. 1).

Gene fusions have been identified as driver mutations in neoplasia for more than three decades, and the insights gained from these studies help shape our understanding of multiple tumorigenesis.^2^ Previous literatures have pointed out several disease-associated structural chromosomal translocations.^3^ For example, the high instability of somatic genome in osteosarcoma, including chromothripsis, chromosomal aneuploidy, and chromosomal rearrangements, could contribute to the production of truncated or fusion proteins, and inhibit many key tumor suppressor genes like *TP53* and *ATRX*, triggering tumor cells migration.^1^ Although many fusion proteins have been validated as effective targets for treating several cancer types in the clinic, there are no such therapies for osteosarcoma. To comprehensively explore this novel area, Kang et al. used TopHat-fusion and SQUID algorithms to screen out some related structural variations in metastatic osteosarcoma. Unlike the prevailing algorithms, which normally filter out intron-related structural variations when searching RNA sequences, this method can preserve exon–intron fusion information, which lays a solid foundation for the following experiment.

Kang et al. first confirmed that the chromosomal translocation-derived aberrant Rab22a was the driver for osteosarcoma lung metastasis in multiple osteosarcoma cell lines and clinical samples. Specially, six fusion transcripts related to *RAB22A* were identified, designated as *RAB22A*-NeoF1-6. All of them contain the first two exons of *RAB22A*, which encode the first 38 amino acids of Rab22a and multiple inverted noncoding regions of chromosome 20. To investigate the role of these transcripts, they were overexpressed in different osteosarcoma cell lines and cell migration and invasion, but not proliferation, were significantly increased. Consistently, authors screened some clinical samples from osteosarcoma patients w/o metastasis and got the same result. Notably, among these six fusions, they revealed that the protein products of *RAB22A*-NeoF1 were more stable and dominant when compared to others. Therefore, they selected Rab22a-NeoF1 for further study. And by using fluorescence in situ hybridization and immunoprecipitation, the specific presence of endogenous Rab22a-NeoF1 in ZOS/ZOS-M cell lines and tumor tissues was determined. Furthermore, via either knockdown or overexpression of Rab22a-NeoF1 in osteosarcoma cell lines and tumor tissues, they verified that Rab22a-NeoF1 could promote tumor migration and invasion, which might be a potential therapeutic target in patients with metastatic osteosarcoma.

The next step of experiment was to investigate how Rab22a-NeoF1 drives metastasis. To explore the Rab22a-NeoF1 protein interactome, authors performed tandem affinity purification (TAP) with mass spectrometry (TAP–MS), and determined Rho family members as the key downstream factors in metastasis. Then, through coimmunoprecipitation and other techniques, Rab22a-NeoF1 was uncovered to promote osteosarcoma lung metastasis by activating RhoA.

The above data indicated that the activation of RhoA by Rab22a-NeoF1 could be a critical determinant to boost the metastasis in osteosarcoma. The authors next sought to how RhoA is activated by Rab22a-NeoF1. From TAP–MS data and co-transfection results, they showed that Rab22a-NeoF1 constitutively bound to a negatively charged region of SmgGDS-607. Interestingly, this region of SmgGDS has been reported to be crucial for its association with RhoA. Based on that, Kang et al. further revealed that the interaction between SmgGDS-607 and RhoA was notably diminished in the presence of Rab22a-NeoF1, indicating that Rab22a-NeoF1 changes the binding of SmgGDS-607 to RhoA and transfers RhoA into active form.

To further investigate the binding area of Rab22a-NeoF1 and SmgGDS-607, Kang et al. confirmed that amino acids 1–10 of Rab22a-NeoF1 was required for the interaction with SmgGDS-607. Meanwhile, the author team has already reported that the promoting function of Rab22a-NeoF1 is largely dependent on its Lys7 acetylation in osteosarcoma.^4^ Based on that, they focused on some positively charged residues and found that Arg4 and Lys7 of Rab22a-NeoF1 were essential for this interaction during lung metastasis. And when using specific targeting peptides of Rab22a, the interaction between Rab22a-NeoF1 and SmgGDS-607 was abolished, which inhibited lung metastasis and increased the survival time, suggesting a potential therapeutic target for osteosarcoma lung metastasis.

Collectively, through comprehensive analysis of different osteosarcoma cell lines, animal models, and patient samples, Kang et al. discovered that Rab22a-NeoF1/SmgGDS-607/RhoA axis is one of the potential mechanisms in driving tumor metastasis. This paper also highlights the importance to understand the genes formed by fusion of exons and introns, which have been ignored by most analyses. With recent advances in deep-sequencing technologies, diverse gene fusions, along with their functions, have been gradually identified and elucidated. More details to understand the fresh fusions are warranted, especially about the mechanisms of modification, downstream factors they target, and cellular processes they regulate. Previous clinical analysis has demonstrated some fusions in other cancers like breast cancer. Considering that clinical trials targeting on fusion proteins have been successfully applied in other tumors,^5^ this research provides experimental evidence and clinical basis for therapies targeting truncated or fusion proteins against osteosarcoma lung metastasis.
